# The Avian-RUM Block: A Locoregional Technique for Distal Wing Procedures in Birds—An Anatomical Cadaveric Feasibility Study with a Clinical Illustration

**DOI:** 10.3390/ani16020211

**Published:** 2026-01-10

**Authors:** Matteo Serpieri, Giuseppe Bonaffini, Elena Passarino, Margherita de Silva, Giuseppe Quaranta, Mitzy Mauthe von Degerfeld

**Affiliations:** 1 Department of Veterinary Sciences, University of Turin, Largo Braccini 2, 10095 Grugliasco, TO, Italy; matteo.serpieri@unito.it (M.S.); elena.passarino@unito.it (E.P.); giuseppe.quaranta@unito.it (G.Q.); mitzy.mauthe@unito.it (M.M.v.D.); 2 Department of Veterinary Medical Sciences, Alma Mater Studiorum—University of Bologna, Via Tolara di Sopra 50, 40064 Ozzano Emilia, BO, Italy; margiedesilva@gmail.com

**Keywords:** avian anaesthesia, birds, locoregional anaesthesia, peripheral nerve block, RUMM block, wildlife rehabilitation

## Abstract

Anaesthesia in avian species presents significant challenges due to high peri-anaesthetic mortality rates and marked anatomical variability. Multimodal anaesthetic protocols incorporating locoregional techniques are advocated to improve analgesia and minimise systemic drug requirements. While brachial plexus blocks are described for avian wing surgery, the proximity to major vessels and air sacs increases the risk of complications. This study introduces the “Avian-RUM block,” a novel locoregional technique for the distal wing, adapted from the mammalian RUMM block but modified to reflect avian neuroanatomy, which lacks a distinct musculocutaneous nerve. Twelve rock doves (*Columba livia*) and twelve hooded crows (*Corvus cornix*) received transcutaneous injections of a dye solution targeting the radial and median-ulnar nerves at two volumes (0.15 and 0.3 mL/kg). Both species demonstrated high rates of nerve staining and procedural feasibility, with no significant difference in staining length between volumes. An illustrative clinical application of the Avian-RUM block in a domestic goose is also reported to contextualise the cadaveric findings within a multimodal perioperative setting. The results confirm the feasibility of the Avian-RUM block and its potential as a simple, equipment-free technique for regional anaesthesia of the avian wing. These findings provide a foundation for future clinical validation across bird species.

## Simple Summary

Anaesthesia in birds presents significant challenges due to high mortality rates and marked anatomical variability among species. Multimodal anaesthetic protocols, incorporating locoregional techniques, are recommended to improve analgesia and reduce systemic drug requirements. While brachial plexus blocks are commonly described for avian wing surgery, they carry risks of complications, including vascular puncture and injection into air sacs. This study introduces the “Avian-RUM block,” a novel locoregional technique for the distal wing, adapted from the mammalian RUMM block. Unlike its mammalian counterpart, the Avian-RUM block targets only the radial and median-ulnar nerves, reflecting the absence of a distinct musculocutaneous branch in birds. Using cadavers of rock doves and hooded crows, a dye solution was injected under direct transcutaneous visualisation of the nerves through the brachial skin. Both species showed high rates of nerve staining, with no significant differences between low (0.15 mL/kg) and high (0.3 mL/kg) injection volumes, suggesting that small volumes are sufficient for coverage. Minor interspecific differences reflected anatomical depth of the nerves. A clinical application in a domestic goose is also presented to contextualise the findings within a clinical setting. This approach represents a practical, anatomy-based technique that may enhance the effectiveness of avian anaesthesia across diverse settings.

## 1. Introduction

The demand for avian medicine and surgery has increased considerably in recent years, encompassing both companion birds and free-living wildlife. Consequently, veterinarians are more frequently required to anaesthetise birds for diagnostic and surgical procedures [1,2]. Anaesthesia in avian species presents distinct challenges due to the wide morphological and physiological diversity across taxa, including marked variations in respiratory anatomy, metabolic rate, and thermoregulation. These factors contribute to a relatively narrow margin of safety compared with mammals, resulting in reported anaesthetic-related mortality rates as high as 3.9%, with even higher incidences in compromised or debilitated individuals [3,4,5]. Additional peri-anaesthetic risks include rapid hypothermia, airway obstruction, inadequate ventilation, regurgitation, and prolonged recovery, all of which can significantly impact survival, particularly in small-bodied or stressed avian patients [5].

To mitigate these risks, multimodal anaesthetic protocols are increasingly advocated, incorporating locoregional techniques to reduce systemic drug requirements and their associated adverse effects. Such approaches enhance intra- and postoperative analgesia, improve cardiovascular and respiratory stability, and promote faster recoveries [6]. However, in avian species, the routine implementation of peripheral nerve blocks remains limited. This is largely attributable to interspecific anatomical variability, a paucity of detailed, species-specific neuroanatomical data, and a scarcity of validated, standardised techniques [7].

Among the available locoregional methods, the brachial plexus block is the most commonly described for avian wing surgery. It can be performed using blind anatomical landmarks, ultrasound guidance, or nerve stimulation [8]. Several cadaveric and clinical investigations have evaluated this technique in birds [9,10,11,12,13,14], demonstrating its potential while also highlighting significant limitations, such as anatomical variability, the small calibre of the nerves, restricted spread of the injectate, and the need for species- and size-specific volume adjustments. Reported complications include vascular puncture, incomplete blockade, or inadvertent injection into the coelomic cavity or air sacs, all of which may have serious consequences [8,15].

In mammals, an alternative technique—the RUMM block—targets the radial, ulnar, median, and musculocutaneous nerves at the brachial level to provide anaesthesia and analgesia distal to the elbow. This approach minimises proximity to the thorax and reduces the risk of trauma to adjacent structures [16]. Although not yet reported in birds, its adaptation could prove both feasible and clinically useful for surgical procedures involving the distal wing. Anatomical studies indicate that the avian forelimb is primarily innervated by the radial and median-ulnar nerves, with no distinct musculocutaneous nerve as observed in mammals [17,18,19]. Furthermore, the avian brachial skin is thin and lacks significant subdermal tissue [20,21], and feather removal may allow direct visualisation of neurovascular structures through the skin, enabling a simplified, equipment-free approach targeting the radial and median-ulnar nerves.

The present cadaveric study investigates this adapted technique, herein termed the “Avian-RUM” block, using two clinically relevant avian species commonly encountered in wildlife rehabilitation: the rock dove (*Columba livia*) and the hooded crow (*Corvus cornix*) [22,23].

The objectives were

1. to compare the longitudinal and circumferential spread of injectate within each species at two volumes (0.15 mL/kg and 0.3 mL/kg);
2. to evaluate and compare the procedural feasibility between species using a structured scoring system;
3. to provide an illustrative clinical application of the Avian-RUM block within a multimodal perioperative anaesthetic protocol in a domestic goose (*Anser anser domesticus*). This clinical illustration is intended solely to contextualise the anatomical feasibility findings and does not constitute a formal assessment of analgesic efficacy.

## 2. Materials and Methods

### 2.1. Animals

Twelve adult rock doves (*Columba livia*, 24 wings) and twelve adult hooded crows (*Corvus cornix*, 24 wings) were included in the study. The sample size was determined based on comparable previous studies [9,14,24]. All subjects were cadavers of birds that had been humanely euthanised due to medical conditions unrelated to the present research. The animals were part of the clinical caseload of the Centro Animali Non Convenzionali (CANC), the unit dedicated to the treatment of exotic species and the rehabilitation of wildlife at the Veterinary Teaching Hospital of the University of Turin. These two species were selected to represent the orders Columbiformes and Passeriformes, which are among the most frequently admitted to wildlife rehabilitation centres in Italy and across Europe [23,25].

In accordance with Italian Law 157/1992, which establishes state ownership of all wildlife, authorisation for clinical activities and the publication of data concerning these animals was obtained through the “Progetto Salviamoli Insieme” agreement between the Metropolitan City of Turin (Italy) and the Department of Veterinary Sciences, University of Turin.

All procedures described below were performed immediately following euthanasia; therefore, no cadavers required freezing prior to use. Each subject received an injection of a dye solution targeting the radial and median-ulnar nerves (see Section 2.2 and Section 2.3). For each bird, one wing was randomly allocated (www.randomizer.org; accessed on 10 November 2024) to receive a volume of 0.15 mL/kg (Group Low), while the contralateral wing received 0.3 mL/kg (Group High).

### 2.2. Radial Nerve Block

**Figure 1:**
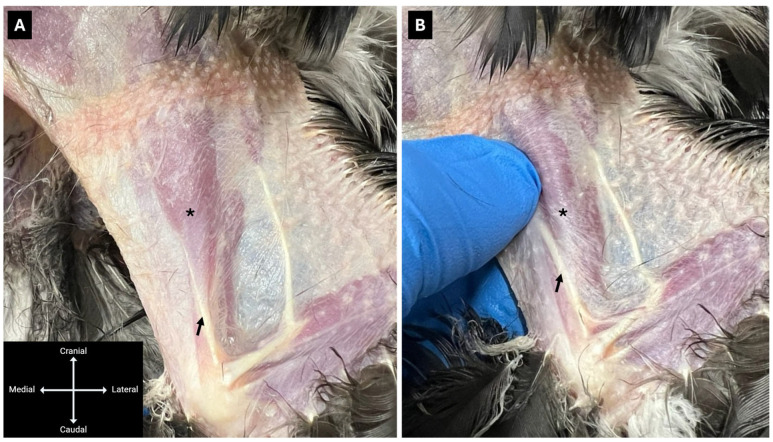
Lateral view of the wing of a hooded crow (*Corvus cornix*) in sternal recumbency. (**A**) The radial nerve (arrow) is visible beneath the *m. deltoideus major* (asterisk). (**B**) Gentle mediolateral displacement of the *m. deltoideus major* (asterisk) with a thumb facilitates transcutaneous visualisation of the radial nerve (arrow).

**Figure 2:**
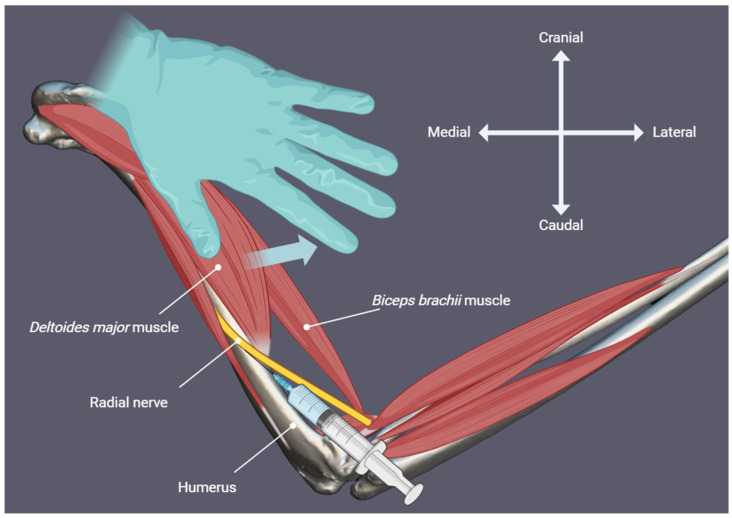
Lateral view. Schematic illustration of the relevant anatomy for performing a radial nerve block in an avian subject, highlighting the course of the radial nerve and the injection site.

With the bird positioned in sternal recumbency, wings extended laterally and pelvic limbs extended caudally, the feathers over the dorsolateral aspect of the humerus were plucked to permit transcutaneous visualisation of the radial nerve at the mid-humeral level, distal to the *m. deltoideus major*. When necessary, the muscle was gently pushed mediolaterally with a thumb to improve visualisation of the radial nerve (Figure 1). A 25-gauge hypodermic needle was inserted at the mid-humerus adjacent to the nerve over the bone (Figure 2), and a 3:1 mixture of 2% lidocaine hydrochloride (Lidocaina 20 mg/mL, Ecuphar Italia S.r.l., Milan, Italy) and 1% methylene blue (Blu di Metilene S.A.L.F. 1%, S.A.L.F. S.p.A., Cenate Sotto, BG, Italy) was injected. Injection volumes were 0.15 mL/kg (Group Low) for one wing and 0.3 mL/kg (Group High) for the contralateral wing (see Section 2.1).

### 2.3. Median-Ulnar Nerve Block

**Figure 3:**
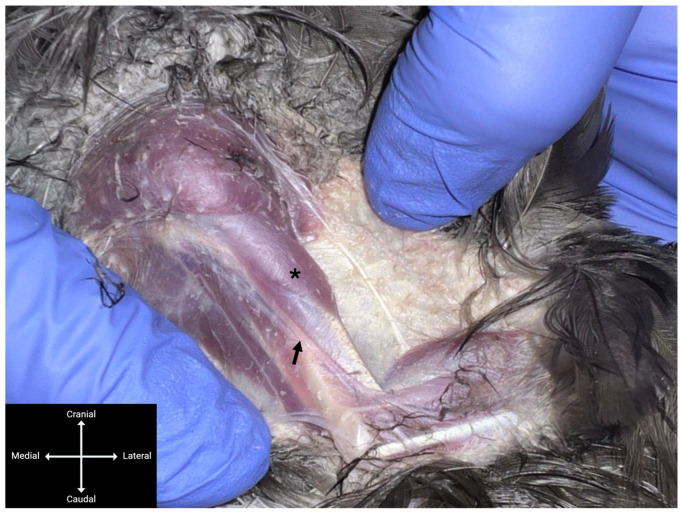
Medial view of the wing of a hooded crow (*Corvus cornix*) in dorsal recumbency. The median-ulnar nerve (arrow) is visible beneath the *m. biceps brachii* (asterisk). Gentle mediolateral displacement of the muscle with a thumb facilitates visualisation of the nerve. In this image, traction was applied to the overlying skin rather than directly to the muscle to enhance exposure.

**Figure 4:**
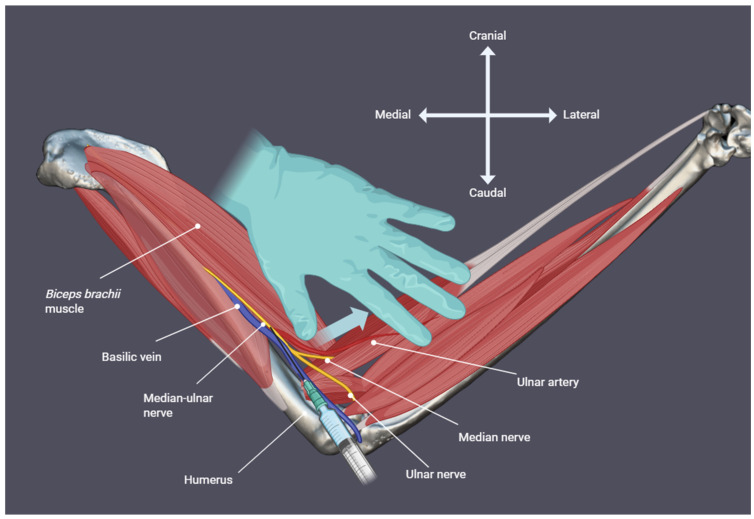
Medial view. Schematic illustration of the relevant anatomy for performing the median-ulnar nerve block in an avian subject, showing the course of the nerve and the recommended injection site. To enhance visualisation of the nerve, the *m. biceps brachii* is gently retracted mediolaterally, allowing identification of the median-ulnar nerve at the distal brachium, lateral to the basilic vein and immediately proximal to its division into the median and ulnar branches.

Each subject was placed in dorsal recumbency, with the wings gently extended laterally and the pelvic limbs positioned caudally. The feathers covering the medial brachial region were plucked to permit transcutaneous visualisation of the underlying neurovascular structures. When necessary, the m. biceps brachii was gently retracted mediolaterally with a thumb to improve visualisation of the median-ulnar nerve (Figure 3), located lateral to the basilic vein and proximal to its division into the median and ulnar branches (Figure 4). The same dye mixture described above was injected, with the needle tip placed in contact with the nerve while carefully avoiding vascular puncture. The same injection volumes described in Section 2.2 were used.

### 2.4. Dissection Procedure and Assessment of Nerve Staining

**Figure 5:**
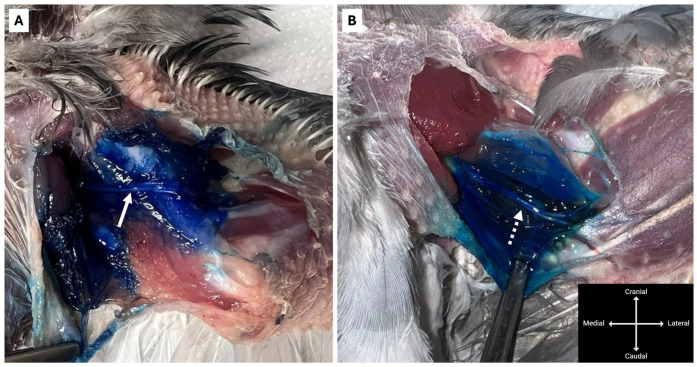
Dissection demonstrating the spread of the injected dye mixture in a rock dove (*Columba livia*). (**A**) Lateral view showing the radial nerve (solid arrow). (**B**) Medial view showing the median-ulnar nerve (dashed arrow).

Immediately after completing the injection procedures, all cadavers underwent dissection to evaluate the spread of the injected mixture (Figure 5).

For the radial nerve block, a skin incision and blunt dissection was made over the humerus to expose the underlying structures. The distal border of the *m. deltoideus major* was incised and reflected proximally to expose the radial nerve. The same procedure was performed on the contralateral wing.

For the median-ulnar nerve, the skin over the neurovascular bundle of the brachium was incised. The distal border of the *m. biceps brachii* was incised and reflected proximally to expose the median-ulnar nerve, which was then gently separated from adjacent vascular structures. The same procedure was repeated on the contralateral wing.

In all subjects, the longitudinal extent (mm) of nerve staining was measured using a calliper, and the completeness of circumferential staining was also assessed. These evaluations were used to assign a dedicated score (see Section 2.5).

### 2.5. Scoring System

For each nerve, a specific scoring system was developed to evaluate visualisation, execution, staining, and feasibility, assigning a score 0 (poor), 1 (good), or 2 (excellent) for each parameter.

Regarding nerve visualization, the following criteria were applied: (0) visualisation of the nerve was difficult even after displacement of the surrounding structures; (1) the nerve was clearly visible after displacement of the adjacent structures; (2) immediate and clear visualisation of the nerve.

For the execution of the block, three parameters were considered: (A) completion of the procedure within 30 s (from feather plucking to injection); (B) visibility of the needle tip during the injection; (C) clear visualisation of the spread of the dye solution around the nerve during the injection. A score of 0, 1, or 2 was assigned when at least one, two, or all three of these parameters were met, respectively.

The staining of the nerve was assessed according to criteria modified from Hon et al. [24]: (0) no staining was visible on the nerve; (1) staining was non-circumferential or <10 mm longitudinally; (2) staining was circumferential and ≥10 mm.

Finally, feasibility was determined based on the previous parameters. A score of 0, 1, or 2 was assigned when at least one, two, or all three of the following criteria were met, respectively: (A) visualisation score ≥ 1; (B) execution score ≥ 1; (C) staining score ≥ 1.

For clarity, the qualitative descriptors “poor”, “good”, and “excellent” were used to facilitate interpretation of the numerical 0–2 scoring system and do not represent success or failure of the technique per se. In this context, a score of “good” (1) indicates an acceptable level of performance for the assessed parameter, while “excellent” (2) indicated optimal conditions in terms of ease of execution, visualisation, and assessment of injectate spread.

### 2.6. Statistical Analysis

Data management and statistical analyses were performed using Microsoft Excel (Microsoft 365, 2024; Microsoft Corp., Washington, DC, USA) and R (version 4.3.2; R Foundation for Statistical Computing, Vienna, Austria). The Shapiro–Wilk test indicated that continuous variables were not normally distributed (*p* < 0.05).

A two-tailed Wilcoxon signed-rank test was applied to compare the longitudinal staining length (mm) between Group Low and Group High for each species. Fisher’s exact test was used to compare the categorical scores obtained for the radial and median-ulnar nerve blocks between species, as well as the fulfilment of the execution and feasibility criteria. Statistical significance was set at *p* < 0.05.

## 3. Results

### 3.1. Cadaveric Study Results

Data are expressed as median and interquartile range (IQR). The body weight of the rock doves was 245 g (205–272 g), whereas that of the hooded crows was 400 g (280–440 g). Data regarding the longitudinal staining length for both nerve blocks are presented in Table 1. The results of the scoring system for the radial nerve block are shown in Table 2, while those for the median-ulnar nerve block are reported in Table 3. The detailed results for the individual criteria contributing to the execution and feasibility scores for both nerve blocks are summarized in Table 4.

### 3.2. Clinical Application of the Avian-RUM Block in a Domestic Goose

A six-year-old, 5 kg male goose was presented with a rounded, lobulated, firm mass (8 × 10 cm) involving the left carpus. The lesion had been present for approximately three months and had progressively enlarged. Given the extent of the lesion and the animal’s domestic status, partial wing amputation was proposed.

Based on clinical examination, the goose was classified ASA II and hospitalised overnight for acclimatisation prior to surgery. Following three hours of fasting, intramuscular (IM) ketamine (4 mg/kg, Lobotor^®^, 100 mg/mL, Acme S.r.l., Corte Tegge-Cavriago, RE, Italy), medetomidine (80 µg/kg, Dormisan^®^, 1 mg/mL, Azienda Terapeutica Veterinaria S.r.l., Milan, Italy), and alfaxalone (0.4 mg/kg, Alfaxan^®^ Multidose, 10 mg/mL, Zoetis Italia S.r.l., Rome, Italy) were administered. After 10 min, a 22G intravenous (IV) catheter (Delta Ven^®^, Delta Med S.p.A., Viadana, MN, Italy) was placed in the right medial metatarsal vein, and propofol (Proposure, 10 mg/mL, Boehringer Ingelheim Animal Health Italia S.p.A., Noventana, PD, Italy) was administered to effect (final dose: 3 mg/kg) to allow intubation with a 5.0 mm silicone Cole endotracheal tube.

Anaesthesia was maintained with isoflurane set at 1.5% on the vaporizer (IsoFlo^®^, Zoetis Italia S.r.l., Rome, Italy) in 100% oxygen using a non-rebreathing system (Bain coaxial breathing system, Intersurgical, Wokingham, Berkshire, UK). In case of apnoea, manual-assisted ventilation was provided at 12 breaths/minute. The following parameters were monitored using a multiparameter monitor (Infinity Delta^®^, Dräger Italia S.p.A., Corsico, MI, Italy): heart and respiratory rates (HR, RR), peripheral saturation of arterial haemoglobin with oxygen (SpO_2_, clip placed on a digit of the hindlimb), end-tidal partial pressure of carbon dioxide (EtCO_2_), and oesophageal temperature (T°). Non-invasive arterial blood pressure was attempted using an oscillometric method with the cuff positioned at the level of the distal tibiotarsus; however, consistent and reliable measurements could not be obtained intra-operatively. Lactated Ringer’s solution (Baxter S.p.A., Rome, Italy) was infused IV at 5 mL/kg/hour.

After induction of general anaesthesia and prior to surgical preparation, the Avian-RUM block was performed following the same anatomical landmarks and technical approach described in the cadaveric study. A 25-gauge hypodermic needle was inserted adjacent to the nerves, and 0.15 mL/kg of a 1:1 mixture of 1% ropivacaine hydrochloride Ropivacaina Cloridrato S.A.L.F., 10 mg/mL; S.A.L.F. S.p.A, Cenate Sotto, BG, Italy) and 2% lidocaine hydrochloride Lidocaina 2%, 20 mg/mL, Ecuphar Italia S.r.l., Milan, Italy) was injected *per* nerve. For both nerve blocks, the local anaesthetic was injected smoothly, without noticeable resistance, and only after a negative aspiration test —defined as the absence of blood upon gentle syringe aspiration—confirmed the needle was not intravascular. For the median-ulnar nerve block, the bird was positioned gently in dorsal recumbency only after confirming stable ventilatory parameters. In this case, localised vasodilation of the basilic vein developed within two minutes, without bleeding or haematoma formation.

Intra-operative rescue analgesia with ketamine (0.5 mg/kg IV) was planned in the event of a 20% increase in HR or RR. Partial wing amputation at the elbow joint was performed, with a total surgical duration of 74 min. No clinically relevant changes in monitored physiological parameters were observed, and no rescue analgesia was required. Intraoperative values [median (range)] were HR 101 (92–106) beats/minute, RR 24 (22–28) breaths/minute, SpO_2_ 97 (95–98)%, EtCO_2_ 45 (42–52) mmHg, and T° 39.9 (39.7–41.1) °C. Manual-assisted ventilation was needed only after propofol administration (1 min), after which spontaneous breathing resumed. Vasodilation at the site of injection resolved spontaneously during the procedure.

Ninety-three minutes after medetomidine administration, atipamezole (0.5 mg/kg IM; Sedastop^®^, 5 mg/mL, Ecuphar Italia S.r.l., Italy) was administered for reversal. Postoperative care included intramuscular administration of meloxicam (0.5 mg/kg; Meloxidyl^®^, 5 mg/mL, Ceva Salute Animale S.p.A., Milan, Italy) and marbofloxacin (10 mg/kg; Marbocyl 10%, Vétoquinol Italia S.r.l., Forlì, FC, Italy), which were continued once daily (q24h) for seven days. The goose was extubated upon the onset of spontaneous movement. Recovery was uneventful, with spontaneous feeding and defaecation resuming immediately after regaining posture and ambulation, 15 min after atipamezole administration.

Post-operative pain was assessed through behavioural observation, as no validated pain assessment scales are currently available for geese [26]. The bird showed normal posture, appetite, and social interaction, with no signs of discomfort on palpation of the wing stump. The goose was discharged two days postoperatively for continued home care. At follow-up examination two months after surgery, complete healing of the surgical site was observed.

## 4. Discussion

This study demonstrates that both the radial and median-ulnar nerve blocks can be successfully performed in the rock dove and the hooded crow using transcutaneous visualisation in cadaveric specimens, achieving generally high rates of circumferential staining and procedural feasibility.

The absence of a significant difference in staining length (Table 1) between the lower and higher injectate volumes (0.15 vs. 0.3 mL/kg) suggests that, within this range, the spread of the injectate is likely influenced more by anatomical factors—such as fascial planes, connective tissue density, nerve depth, and muscle–bone relationships—than by the absolute volume administered. Consequently, the lower volume (0.15 mL/kg) may be considered adequate, as it consistently produced circumferential staining and longitudinal nerve coverage exceeding 10 mm. This finding aligns with the cadaveric study by Micieli et al. [13] on brachial plexus block in common kestrels (*Falco tinnunculus*), which proposed that a staining length greater than 0.6 mm could be sufficient to cover at least three nodes of Ranvier, thereby ensuring an effective nerve block [27,28]. In that study, however, larger dye volumes were used (0.5 mL/kg per wing). In contrast, administering 0.15 mL/kg per nerve (radial and median-ulnar) in the present work would result in a total of 0.3 mL/kg per wing—thus achieving effective staining with a lower total injectate volume. This value also remains below those reported in previous cadaveric and clinical studies of avian brachial plexus blocks [9,11,12,14]. However, the feasibility of using 0.15 mL/kg per nerve was confirmed only in the species studied—rock doves and hooded crows. When applying this dosage to smaller avian species, practical considerations must be made regarding injectability and measurement precision. If the resulting volume is too small to measure accurately (e.g., <0.02 mL), a larger volume should be considered, potentially achieved through dilution. Nevertheless, the use of reduced volumes may help to minimise the potential risk of local anaesthetic toxicity, an issue that remains controversial in avian medicine due to the scarcity of clinical studies defining accurate and safe dose ranges [13,29].

In this study, the proximity of the needle tip to the nerve, facilitated by direct visualisation through the skin, represents a potential advantage, allowing small-volume injections and immediate assessment of injectate spread. However, such proximity also entails a risk of iatrogenic nerve injury or venipuncture, particularly when approaching the median-ulnar nerve [6]. Thus, meticulous technique and sound anatomical knowledge are essential to ensure both efficacy and safety. Adjunctive use of nerve stimulation or ultrasound guidance could further enhance accuracy and reduce the incidence of complications [30]. Although such modalities are routinely recommended for performing RUMM blocks in mammals, their advantages may not fully translate to avian patients. Direct transcutaneous visualisation of nerves, vessels, and the needle can already provide sufficient accuracy, while the bulk of the ultrasound probe may complicate manoeuvrability—particularly in small-bodied birds where the target structures are extremely superficial and lie in close proximity to the probe [31]. The direct-visualisation approach described here is also suitable for diverse clinical contexts, including resource-limited settings and wildlife rehabilitation centres, where access to advanced and costly equipment is often restricted. Indeed, one of the main barriers to the widespread adoption of locoregional anaesthesia in exotic and wild species is the limited availability of ultrasound machines or electrical nerve stimulators [7].

From an anatomical standpoint, the radial nerve represents one of the principal terminal branches of the avian brachial plexus and is considered a major contributor to both motor and sensory innervation of the distal wing. Anatomical studies across different avian species indicate that the radial nerve is primarily associated with the extensor musculature of the elbow, carpus and digits, while also providing cutaneous sensory input from the dorsal aspect of the wing and antebrachium [18,32]. On this basis, blockade of the radial nerve at the mid-humeral level, as performed in the present study, would be expected to reduce nociceptive input originating from the dorsal and distal portions of the wing, together with a transient impairment of extension-related motor function distal to the elbow. In this context, the concurrent blockade of the median-ulnar nerve is expected to complement the analgesic effect by targeting additional sensory territories of the distal wing, thereby providing a more comprehensive regional analgesia for procedures performed distal to the elbow. This conceptual approach closely parallels the rationale of the RUMM block in veterinary medicine, where the combined blockade of the radial, ulnar and median nerves is employed to achieve effective analgesia of the distal thoracic limb while avoiding the risks associated with more proximal brachial plexus techniques [15,16]. A similar strategy is also well established in human anaesthesia, where selective distal nerve blocks at the arm or elbow level are used to provide surgical analgesia for procedures of the forearm and hand, while limiting unnecessary proximal motor blockade and associated morbidity [33]. It should be emphasised, however, that these considerations are based on anatomical and physiological evidence rather than on direct functional assessment. Although neuroanatomical and neurophysiological studies support a structured sensory representation of the avian wing at the spinal level [34], the actual sensory and motor effects of radial nerve blockade in live birds remain to be clinically validated. Consequently, the present findings should be interpreted as providing an anatomical rationale for the proposed technique, rather than as definitive proof of its functional efficacy.

To provide clinical context to the anatomical feasibility demonstrated in this cadaveric study, the Avian-RUM block was also applied in a representative clinical case involving a domestic goose undergoing partial wing amputation. In that setting, the technique was incorporated into a multimodal anaesthetic protocol and was associated with stable intra-operative physiological parameters and the absence of behavioural or physiological indicators suggestive of inadequate nociceptive control throughout the procedure. In this case, the lidocaine–ropivacaine combination was selected to balance rapid onset and prolonged duration of action [10,35]. However, it must be acknowledged that pharmacokinetic and pharmacodynamic data for local anaesthetics in birds remain limited. Consequently, the clinical observations reported should be interpreted as illustrative rather than confirmatory of block efficacy. From a practical point of view, the ease of identifying the injection sites without the need for ultrasound guidance or nerve stimulation allows the Avian-RUM block to be performed rapidly and integrated into the anaesthetic workflow without prolonging anaesthesia time. This represents a relevant practical advantage in avian patients, in whom minimising procedural duration is a key component of peri-anaesthetic safety.

Regarding interspecific differences, hooded crows exhibited lower visualisation and execution scores for both the radial and median-ulnar nerve blocks. Several anatomical factors may account for this variation. The avian brachial plexus and its branches are known to display a degree of interspecific variability in their origin, course, and branching patterns [17,18,19,36]. Such differences may complicate the reliable identification of the median-ulnar trunk, resulting in a less favourable needle approach angle, greater depth of target structures, or closer proximity to major vessels. In the Authors’ experience, hooded crows tend to be more robust than rock doves, with more developed flight musculature and deeper brachial neurovascular bundles. Consequently, in this species it was often necessary to adapt the approach to the median-ulnar nerve (Table 3) by gently retracting the *m. biceps brachii* mediolaterally to improve nerve visualisation. Also, the radial nerve appeared more distinct along its course over the humerus in rock doves, whereas in hooded crows it was partially obscured by overlying musculature. This interspecific anatomical variability likely explains the statistically significant differences observed in visualisation and execution scores between the two species for both blocks (Table 2), and those observed for the median-ulnar nerve block in the staining and feasibility scores.

**Figure 6:**
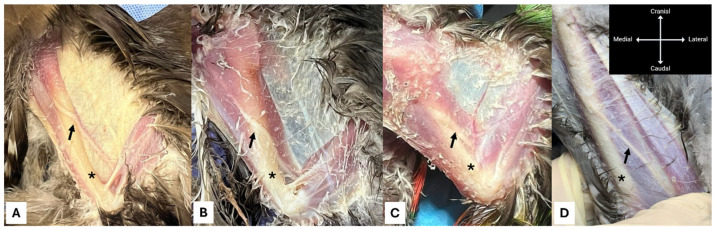
Lateral view of the wing in various avian species positioned in sternal recumbency, showing the radial nerve (arrow). The humerus is indicated by an asterisk. (**A**) Common buzzard (*Buteo buteo*); (**B**) Eurasian goshawk (*Astur gentilis*, formerly *Accipiter gentilis*); (**C**) Rosy-faced lovebird (*Agapornis roseicollis*); (**D**) Grey heron (*Ardea cinerea*).

**Figure 7:**
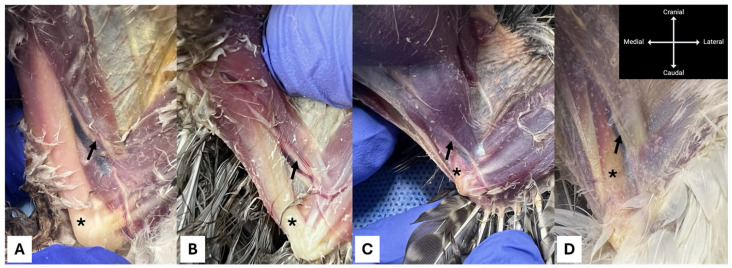
Medial view of the wing in various avian species positioned in dorsal recumbency, showing the median-ulnar nerve (arrow). The humerus is indicated by an asterisk. (**A**) Eurasian sparrowhawk (*Accipiter nisus*); (**B**) Common kestrel (*Falco tinnunculus*); (**C**) European green woodpecker (*Picus viridis*); (**D**) Mallard (*Anas platyrhynchos*).

From a clinical standpoint, our findings highlight the importance of adapting the technique to species-specific morphological characteristics. Although no published data are currently available, in the Authors’ experience, direct transcutaneous visualisation of the radial nerve is generally easier in larger avian species (e.g., buzzards, herons) (Figure 6). Conversely, the medial neurovascular bundle tends to be more readily identifiable in smaller birds (e.g., woodpeckers, kestrels) (Figure 7), while it may be more difficult to visualise in species with relatively thicker skin, such as aquatic birds (e.g., geese). The high feasibility observed in rock doves suggests that smaller, relatively thin-winged species may represent suitable models for the application and further refinement of the Avian-RUM block. In clinical practice, the Avian-RUM block may be indicated for surgical procedures involving the wing distal to the elbow, such as the treatment of fractures of the radius, ulna, or metacarpal bones—conditions frequently encountered in birds admitted to wildlife rehabilitation centres [37,38]. Additional potential applications include the surgical treatment of neoplastic or traumatic lesions affecting the distal wing, which are relatively common in certain species, particularly psittacines [39]. When applied in such contexts, the Avian-RUM block may enhance perioperative analgesia, potentially allowing for reduced doses of systemic injectable or inhalant anaesthetics and consequently minimising their associated adverse effects [40].

From a practical standpoint, patient positioning should also be considered. The described technique requires two separate injections—lateral and medial—performed with the bird placed in sternal and dorsal recumbency, respectively. Although dorsal recumbency may impair respiratory mechanics in avian patients [1,5], the median-ulnar nerve approach is relatively quick to perform (Table 4), thereby limiting the duration of this position. As with any anaesthetic procedure, appropriate clinical and instrumental monitoring of the patient’s general anaesthesia is strongly recommended to ensure safety and efficacy [5,40].

The limitations of this study include the use of only two species, which cannot, of course, be considered representative of the entire class *Aves*. Nevertheless, despite some degree of interspecific anatomical variability [36], the components of the brachial plexus appear to be relatively conserved across different avian taxa, which may facilitate the application of the Avian-RUM block to other species, provided that minor anatomical adaptations are made. Although this study included only two species, it can be hypothesised that the anatomical features observed in rock doves and hooded crows are broadly representative of their respective orders, Columbiformes and Passeriformes. Therefore, the described procedures may also be successfully performed in other avian species, potentially extending to different taxonomic groups (Figure 6 and Figure 7). Another limitation is the use of cadaveric specimens, which precludes any functional (sensory or motor) assessment of block efficacy and may not fully replicate the tissue compliance and physiological conditions of live birds. To minimise these limitations, however, all procedures were performed immediately after euthanasia, ensuring that tissue integrity and consistency were preserved as much as possible. Furthermore, although dye spread represents a useful proxy for injectate diffusion, clinical outcomes in vivo may differ due to factors such as blood circulation, metabolism, and the dynamic characteristics of living tissues, as well as physicochemical differences between local anaesthetics and the dye–diluent combinations employed in this study [41,42]. The inclusion of a single clinical application does not alter the fundamentally anatomical nature of the present work and does not allow conclusions to be drawn regarding analgesic efficacy. Rather, it serves to contextualise the proposed technique within a real-world perioperative setting and to illustrate its potential integration into multimodal anaesthetic protocols in avian patients.

In the context of avian medicine, where marked interspecific anatomical and physiological variability exists across an entire taxonomic class, cadaveric feasibility studies represent a particularly important and legitimate step in the development of locoregional anaesthetic techniques. Rather than aiming to demonstrate clinical efficacy, the present study was designed to provide an anatomical and technical framework for the Avian-RUM block, enabling reproducibility, volume optimisation, and risk assessment across species. By establishing procedural feasibility in two clinically relevant avian models, this work is intended to facilitate and encourage subsequent in vivo clinical and experimental studies evaluating analgesic efficacy and safety in target species of interest, which may vary substantially in their anatomical features and clinical response.

## 5. Conclusions

In conclusion, this anatomical study demonstrates the feasibility of the Avian-RUM block in two avian species, providing evidence that both the radial and median-ulnar nerves can be effectively targeted through direct transcutaneous visualisation. The results underscore the importance of adapting the technique to species-specific morphological features and suggest that low-volume injections may be sufficient to achieve adequate nerve coverage while minimising potential complications. Clinically, this approach may offer a simple and equipment-free option for providing regional anaesthesia in surgical procedures involving the distal wing, improving perioperative analgesia and reducing systemic anaesthetic requirements. Overall, these findings provide a valuable anatomical and methodological foundation for the future refinement and clinical validation of loco-regional anaesthesia protocols in birds, supporting safer and more effective analgesic strategies in avian medicine and wildlife care.

## Figures and Tables

**Table 1:** Longitudinal staining length [median (interquartile range)] for the radial and median-ulnar nerves block in twelve rock doves (*Columba livia*) and twelve hooded crows (*Corvus cornix*), following injection of Low (0.15 mL/kg) and High (0.3 mL/kg) volumes. Significance was set at *p* < 0.05.

	Radial Nerve Stain (mm)			Median-Ulnar Nerve Stain (mm)		
	Low	High	*p* Value	Low	High	*p* Value
Rock doves	17 (15–19)	22 (19–23)	0.13	14 (14–17)	21 (19–23)	0.25
Hooded crows	24 (21–28)	23 (22–29)	0.36	21 (15–26)	20 (18–23)	0.51

**Table 2:** Visualisation, execution, staining, and feasibility scores [number (%)] for the radial nerve block in twelve rock doves (*Columba livia*) and twelve hooded crows (*Corvus cornix*). Significance (*) was set at *p* < 0.05.

Radial Nerve—Scores				
		Rock Doves	Hooded Crows	*p* Value
Visualisation	Poor (0)	2 (8.3%)	0 (0%)	2.93 × 10^5^ *
	Good (1)	12 (50.0%)	24 (100%)	
	Excellent (2)	10 (41.7%)	0 (0%)	
Execution	Poor (0)	0 (0%)	0 (0%)	0.03 *
	Good (1)	4 (16.7%)	12 (50.0%)	
	Excellent (2)	21 (87.5%)	12 (50.0%)	
Staining	Poor (0)	0 (0%)	0 (0%)	1.00
	Good (1)	1 (4.2%)	1 (4.2%)	
	Excellent (2)	23 (95.8%)	23 (95.8%)	
Feasibility	Poor (0)	0 (0%)	0 (0%)	0.61
	Good (1)	2 (8.3%)	1 (4.2%)	
	Excellent (2)	22 (91.7%)	23 (95.8%)	

**Table 3:** Visualisation, execution, staining, and feasibility scores [number (%)] for the median-ulnar nerve block in twelve rock doves (*Columba livia*) and twelve hooded crows (*Corvus cornix*). Significance (*) was set at *p* < 0.05.

Median-Ulnar Nerve—Scores				
		Rock Doves	Hooded Crows	*p* Value
*Visualisation*	Poor (0)	0 (0%)	11 (45.8%)	8.65 × 10^5^ *
	Good (1)	16 (66.7%)	11 (45.8%)	
	Excellent (2)	18 (33.3%)	2 (8.3%)	
*Execution*	Poor (0)	0 (0%)	1 (4.2%)	8.30 × 10^4^ *
	Good (1)	6 (25.0%)	17 (70.8%)	
	Excellent (2)	21 (75.0%)	6 (25.0%)	
*Staining*	Poor (0)	1 (4.2%)	0 (0%)	0.01 *
	Good (1)	0 (0%)	7 (29.2%)	
	Excellent (2)	23 (95.8%)	17 (70.8%)	
*Feasibility*	Poor (0)	1 (4.2%)	0 (0%)	2.87 × 10^4^ *
	Good (1)	1 (4.2%)	12 (50.0%)	
	Excellent (2)	21 (87.5%)	12 (50.0%)	

**Table 4:** Fulfilment of the criteria for the execution and feasibility scores [number (%)] for the median-ulnar nerve block in twelve rock doves (*Columba livia*) and twelve hooded crows (*Corvus cornix*). For the execution score, the criteria were as follows: (A) completion of the procedure within 30 s (from feather plucking to injection); (B) visibility of the needle tip during the injection; (C) clear visualisation of the spread of the dye solution around the nerve during the injection. For the feasibility score, the criteria were as follows on the basis of the other scores: (A) visualisation score ≥ 1; (B) execution score ≥ 1; (C) staining score ≥ 1. Significance (*) was set at *p* < 0.05.

		Execution Score Criteria			Feasibility Score Criteria		
		Rock Doves	Hooded Crows	*p* Value	Rock Doves	Hooded Crows	*p* Value
Radial nerve	A	24 (100%)	24 (100%)	1.00	22 (91.7%)	24 (100%)	0.25
	B	22 (91.7%)	22 (91.7%)	1.00	24 (100%)	24 (100%)	1.00
	C	22 (91.7%)	14 (58.3%)	0.02 *	24 (100%)	24 (100%)	1.00
Median-ulnar nerve	A	24 (100%)	24 (100%)	1.00	24 (100%)	13 (54.2%)	8.57 × 10^5^ *
	B	20 (83.3%)	18 (75.0%)	0.73	23 (95.8%)	23 (95.8%)	1.00
	C	22 (91.7%)	11 (45.8%)	5.77 × 10^4^ *	23 (95.8%)	24 (100%)	0.49

## Data Availability

Further specific data regarding each animal can be requested from the Authors.
